# Assessment of intrahepatic cholangiocarcinoma with LI-RADS in the high-risk population: MRI diagnosis and postoperative survival

**DOI:** 10.1186/s40644-025-00860-6

**Published:** 2025-03-26

**Authors:** Ruofan Sheng, Beixuan Zheng, Yunfei Zhang, Chun Yang, Dong Wu, Jianjun Zhou, Mengsu Zeng

**Affiliations:** 1 Department of Radiology, Zhongshan Hospital, Fudan University, No. 180 Fenglin Road, Xuhui District, Shanghai, 200032 China; 2 Shanghai Institute of Medical Imaging, Shanghai, 200032 China; 3 Department of Radiology, Zhongshan Hospital (Xiamen), Fudan University, No. 668 Jinhu Road, Huli District, Fujian, 361006 Fujian China; 4 Xiamen Municipal Clinical Research Center for Medical Imaging and Xiamen Key Clinical Specialty for Radiology, Xiamen, 361015 China

**Keywords:** Intrahepatic cholangiocarcinoma, Magnetic resonance imaging, LI-RADS, Recurrence-free survival

## Abstract

**Background:**

The precise impact of LI-RADS-defined risk factors on the diagnosis and prognosis of intrahepatic cholangiocarcinoma (iCCA) remains unclear.

**Objective:**

To assess the value of LI-RADS categories and features for iCCA diagnosis, focusing on the diagnostic and prognostic implications of LI-RADS-defined risk factors.

**Methods:**

Totally 214 high risk patients, including 107 surgically-confirmed solitary iCCAs and 107 hepatocellular carcinomas (HCC) from two centers were retrospectively enrolled. Clinical and MRI features based on LI-RADS v2018 were compared, and the performance of targetoid features for discriminating iCCA was evaluated. Recurrence-free survival (RFS) was compared across different pathologic diagnoses and LI-RADS categories. Multivariate Cox analysis was performed to identify the independent risk factors for RFS.

**Results:**

In the LI-RADS defined high-risk patients, iCCAs differed from HCCs in MRI manifestation. The LR-M category enabled the accurate classification of most iCCAs (89/107, 83.2%), achieving high sensitivity (83.2%), specificity (85.1%), and accuracy (84.1%). The optimal diagnostic performance for iCCA was achieved when at least one targetoid appearance was required for LR-M categorization (AUC = 0.828). Although 26.2% iCCAs presented at least one major feature and 15.0% iCCAs were miscategorized as probably or definitely HCC, only one iCCA case was categorized as LR-5. RFS varied according to both pathologic diagnosis (*P* = 0.030) and LI-RADS category (*P* = 0.028), with LI-RADS category demonstrating an independent association with RFS (HR = 1.736, *P* = 0.033).

**Conclusions:**

In high-risk patients, iCCAs frequently exhibit HCC major features, leading to miscategorization as probable HCC. However, the LR-5 category remains highly specific for ruling out iCCA. Furthermore, in high-risk patients with solitary resected iCCA or HCC, LI-RADS category enables the prediction of postsurgical prognosis independently from pathological diagnosis.

**Supplementary Information:**

The online version contains supplementary material available at 10.1186/s40644-025-00860-6.

## Background

Intrahepatic cholangiocarcinoma (iCCA) is a highly lethal hepatobiliary malignancy with a poor prognosis and is the second most common primary liver cancer after hepatocellular carcinoma (HCC) [1]. It is of great importance to distinguish iCCA from HCC due to their distinct treatment strategy and prognosis [2, 3]. Misdiagnosis of iCCA may impact treatment selection and subsequent treatment outcomes.

The Liver Imaging Reporting and Data System (LI-RADS) is an algorithm for standardizing the imaging diagnosis and risk stratification of HCC [4]. It is specifically intended for use in high-risk populations for HCC, primarily adults with chronic hepatitis B or cirrhosis [4]. However, studies have shown that chronic hepatitis B and cirrhosis are also major risk factors for iCCA, particularly in China and other East Asian countries [5–7]. In cirrhotic livers, altered hepatic blood flow can lead to atypical enhancement patterns, making iCCA diagnosis particularly challenging in high-risk patients due to overlapping imaging features with HCC [8–10]. Generally, the LR-4 or 5 categories indicate a high probability of HCC, while the LR-M category is most closely associated with iCCA [4, 8]. However, the precise impact of HCC-related risk factors on the diagnostic performance of LI-RADS for discriminating iCCA from HCC remains unclear. Furthermore, emerging evidence suggested that LI-RADS category may provide prognostic information, with patients categorized as LR-M exhibiting worse outcomes in both iCCA and HCC [11–13]. However, the prognostic significance and relative weight of LI-RADS category in high-risk populations need to be clarified.

The purpose of this study was to assess the value of LI-RADS categories and imaging features—particularly the targetoid LR-M features—for iCCA diagnosis in the high-risk patients from two centers, discriminating from HCC. Additionally, it sought to evaluate the potential prognostic implications of LI-RADS-defined risk factors.

## Materials and methods

### Study population

**Fig. 1:**
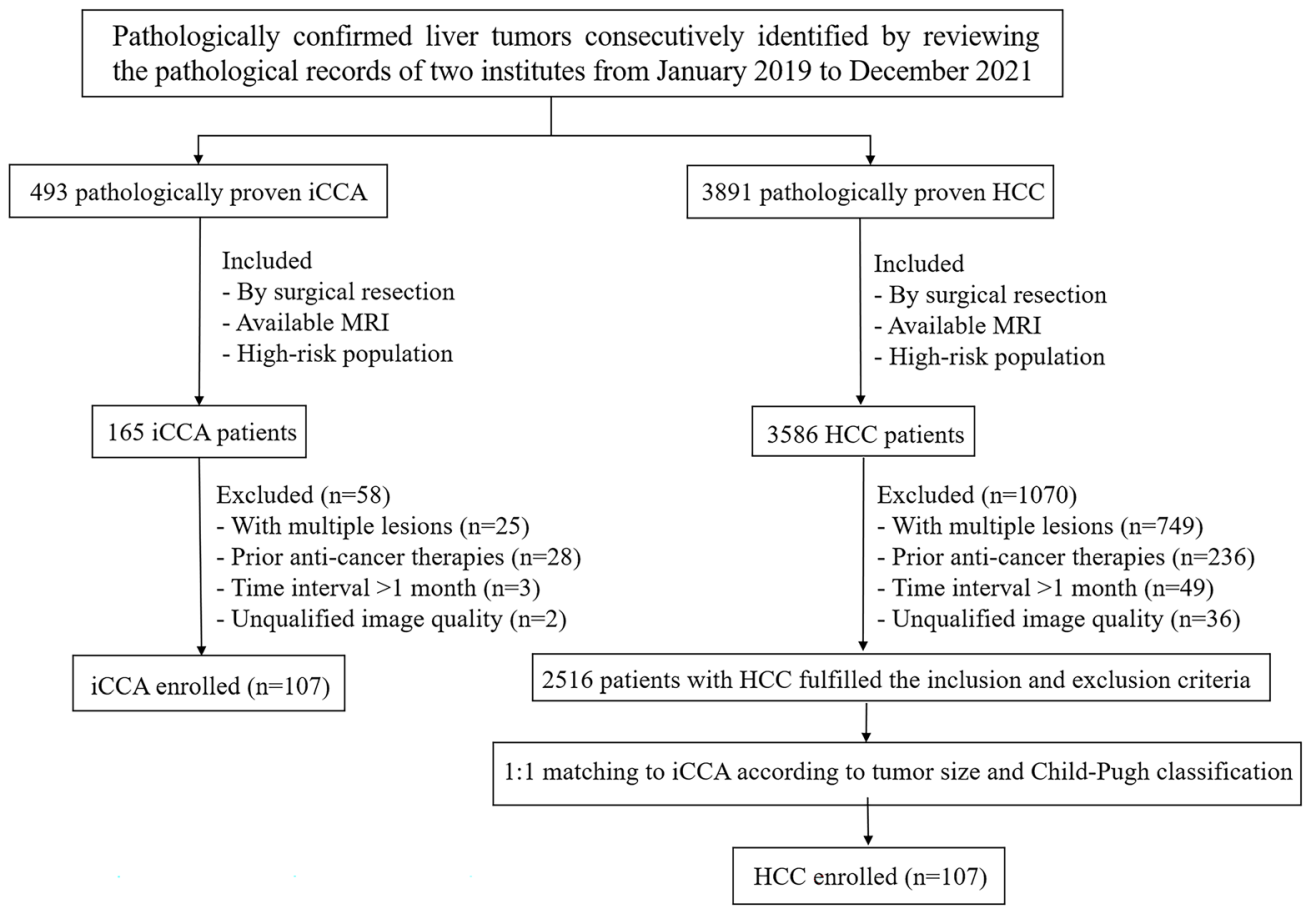
Patients’ inclusion and exclusion flowchart

This retrospective study was approved by the institutional Ethical Review Committee and the requirement for written informed consent was waived. Patients with pathologically-confirmed iCCA were identified within the database of two institutes from January 2019 to December 2021. The inclusion criteria were: (1) pathologic diagnosis by surgical resection; (2) available preoperative contrast-enhanced MRI; (3) high-risk population by LI-RADS v2018 criteria [4] according to prior study [10]. The exclusion criteria were: (1) patients with multiple lesions; (2) prior local-regional or systemic anti-cancer therapies; (3) time interval between MRI and surgery more than 1 month; (4) unqualified image quality with severe artifacts. Patients with surgically proven solitary HCC who fulfilled the inclusion and exclusion criteria above were enrolled by 1:1 matching with iCCA according to tumor size (± 5 mm) and Child-Pugh classification (A, B, C) [14]. Flowchart of the inclusion and exclusion criteria is presented in Fig. 1.

A total of 214 high-risk patients who had surgical resection and preoperative contrast-enhanced MRI were enrolled (107 HCC and 107 ICC; 143 men and 71 women; mean age 57.44 ± 9.99 years, ranges 25.0–80.0 years).

### Image acquisition

All patients were examined with 1.5 or 3.0 Tesla MR scanners (Magnetom Aera, Siemens, Erlangen, Germany; uMR 770, United Imaging Healthcare, Shanghai, China). Routine plain-scan protocols consisted of respiratory-triggered T2-weighted (T2W) fat-suppressed fast spin-echo sequence, T1-weighted (T1W) in-phase and opposed-phase gradient echo sequence, and free-breathing single-shot spin-echo echo-planar diffusion-weighted imaging (DWI) with b values of 0, 50 and 800 s/mm^2^. Contrast-enhanced imaging was performed with breath-hold T1W 3D fat-suppressed gradient-echo sequences, before and after the intravenous administration of gadopentetate dimeglumine (Bayer HealthCare, Berlin, Germany). Contrast was administered at a dose of 0.1 mmol/kg at a rate of 2 mL/s. The arterial phase acquisitions were triggered automatically when contrast media reached the ascending aorta. The portal venous and delay phases were subsequently performed at about 60–70 s and 160–180 s. Detailed scanning parameters are listed in Table S1.

### Image analysis

MR images were independently evaluated by 2 radiologists (with 9 and 18 years of experience in liver MRI) using a picture archiving and communication system (Pathspeed, GE Medical Systems Integrated Imaging Solutions, Prospect, IL, USA). When discrepancies occurred, a consensus was reached after discussion for final decision. The reviewers were aware that the patients had primary liver cancers, but were blinded to all other information, including patients’ clinicopathological results and follow-up information. Cases were randomized to intermix iCCAs and HCCs. Major features, LR-M features, ancillary features favoring HCC in particular and favoring malignancy but not HCC in particular were assessed based on the LI-RADS v2018 [4], and LI-RADS category was assigned. Targetoid appearance on T2-weighted images was defined as a concentric pattern of peripheral hyperintensity and central hypointensity [15]. Tumor size (largest diameter) was measured by the senior reviewer with 18 years of experience in liver MRI.

### Follow-up

One hundred and seventy-five patients were followed up after surgery, among whom 11 patients were censored within 6 months after surgery. Totally 164 patients were regularly followed up for survival analysis. Recurrence was defined as intrahepatic or extrahepatic neoplasms detected. The last follow-up date was set at May 31, 2023. Recurrence-free survival (RFS) was calculated from the date of surgery to the date of tumor recurrence or last follow-up.

### Statistical analysis

All statistical analyses were conducted using the SPSS software (version 26.0). The normality of the data was tested using the Kolmogorov-Smirnov test, and the homogeneity of data was tested using Levene’s method. Interobserver agreement was determined using kappa statistics: 0.00, poor; 0.00-0.20, slight; 0.21–0.40, fair; 0.41–0.60, moderate; 0.61–0.80, substantial and 0.81-1.00, almost perfect [16]. The clinical and MRI findings between iCCA and HCC at risk were compared by the independent *t*-test or Mann-Whitney U test for continuous variables, and the Pearson’s *χ*^*2*^ test or Fisher’s exact test for categorical variables, as appropriate. Univariate and multivariate logistic regression analyses were performed to identify the independent MRI features in discrimination. In order to correct for the multiple comparisons, the Bonferroni correction was applied and a *P*-value threshold of 0.00208 (0.05/24) was used for significance for the univariate analyses in order to keep a family-wise type 1 error rate of 5%. The performance of targetoid features for discrimination was evaluated by the receiver operating characteristic curve analysis. The area under the curve (AUC) values were compared by DeLong’s method. RFS was estimated by using Kaplan-Meier survival curves and compared between different pathologic diagnoses or LI-RADS categories by the log-rank test. A multivariate analysis of independent risk factors related to RFS after surgical resection was conducted by the Cox proportional hazard model with backward selection, including age, sex, pathologic diagnoses, LI-RADS category, serum alpha fetoprotein (AFP) level and cancer antigen 19 − 9 (CA19-9) level. All tests were two-sided, and *P* < 0.05 was considered significant.

## Results

### Demographic characteristics of patients

Baseline demographic characteristics of all study patients are demonstrated in Table 1. Compared to patients with HCC, iCCAs occurred more frequently in patients with older age (*t* = 2.25, *P* = 0.025), normal serum AFP (*χ*^*2*^ = 36.53, *P* < 0.001) and elevated CA19-9 (*χ*^*2*^ = 26.15, *P* < 0.001) levels.

**Table 1:** Demographic characteristics of all study patients

Variable	iCCA (*n* = 107)	HCC (*n* = 107)	*P*
Age (y)^†^	58.96 ± 9.86	55.92 ± 9.94	0.025^*^
Sex Male/Female	65(60.7)/42(39.3)	78(72.9)/29(27.1)	0.059
Risk etiologies			0.119
Chronic HBV infection	96 (89.7)	102 (95.3)	
Cirrhosis (HBV)	63	78	
Cirrhosis (non-HBV)	11 (10.3)	5 (4.7)	
Cryptogenic	3	2	
Alcoholic	1	0	
Hepatitis C virus	1	2	
Schistosome	3	0	
NAFLD	2	1	
Others	1	0	
AFP ≥ 20/<20 ng/mL	13(12.1)/94(87.9)	54(50.5)/53(49.5)	< 0.001^*^
CA19-9 ≥ 37/<37 ng/mL	57(53.3)/50(46.7)	21(19.6)/86(80.4)	< 0.001^*^
CEA ≥ 5/<5 ng/mL	14(13.1)/93(86.9)	11(10.3)/96(89.7)	0.523
TBil > 20.4/≤20.4 µmol/L	7(6.5)/100(93.5)	13(12.1)/94(87.9)	0.159
ALT > 35/≤35 U/L	22(20.6)/85(79.4)	39(36.4)/68(63.6)	0.010^*^
AST > 40/≤40 U/L	16(15.0)/91(85.0)	32(29.9)/75(70.1)	0.009^*^
ALP > 125/≤125 U/L	15(14.0)/92(86.0)	22(20.6)/85(79.4)	0.206
γGGT > 60/≤60 U/L	22(20.6)/85(79.4)	50(46.7)/57(53.3)	< 0.001^*^
Child-Pugh classification A/B/C	101(94.4)/6(5.6)/0(0)	105(98.1)/2(1.9)/0(0)	0.280

Unless otherwise indicated, data are numbers of patients with percentages in parentheses

^†^ Data are means ± standard deviations

^*^P < 0.05

iCCA, intrahepatic cholangiocarcinoma; HCC, hepatocellular carcinoma; HBV, hepatitis B virus; NAFLD, non-alcoholic fatty liver disease; AFP, alpha fetoprotein; CA19-9, cancer antigen 19 − 9; CEA, carcinoembryonic antigen; TBil, total bilirubin; ALT, alanine aminotransferase; AST, aspartate aminotransaminase; ALP, alkaline phosphatase; γGGT, γ-glutamyltransferase

### LI-RADS features and categories of lesions

**Fig. 2:**
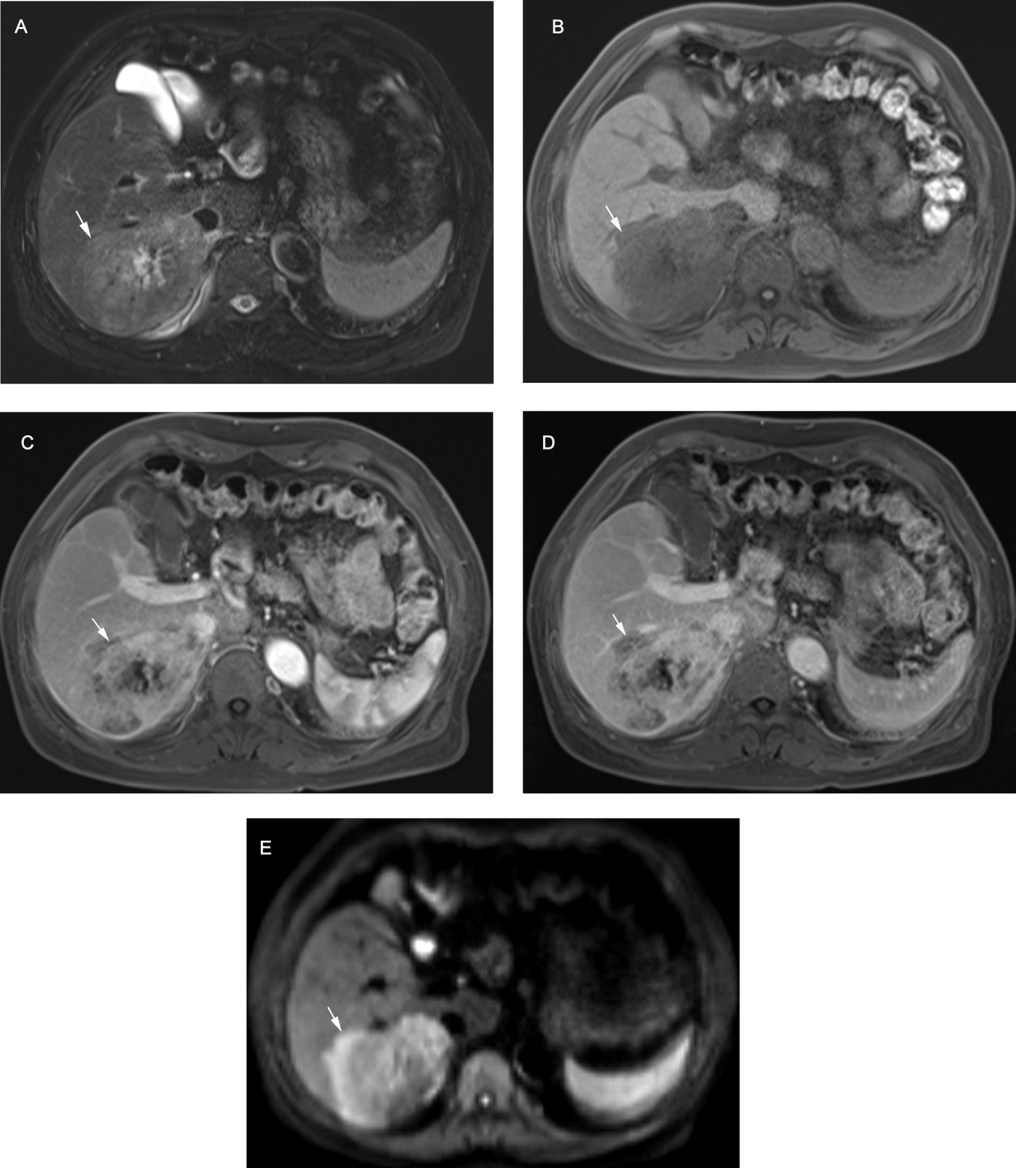
Intrahepatic cholangiocarcinoma in a 74-year-old man (white arrows). (**A**) T2-weighted image shows a mild hyperintense tumor with central necrosis. (**B**) T1-weighted image shows a hypointense tumor. Contrast-enhanced (**C**) arterial phase image shows non-rim hyperenhancement; (**D**) delayed image shows a non-washout (persistent) enhancement pattern. (**E**) Diffusion-weighted image (b = 500 s/mm^2^) shows targetoid restriction. The lesion is categorized as LR-M

The relative frequencies of MRI features including major features, LR-TIV, LR-M, and ancillary features among iCCAs versus HCCs in the LI-RADS high-risk population are summarized in Table 2. LI-RADS major features of non-rim arterial phase hyperenhancement (APHE), non-peripheral washout and enhancing capsule were all significantly more frequent among HCCs (*χ*^*2*^ = 96.97 to 142.27, *P* < 0.001 for all); while a fair portion of iCCAs also showed positive major features, with 28 (26.2%) presented at least one major features (Fig. 2). The LR-M targetoid features (including rim APHE, delayed central enhancement, targetoid diffusion restriction, as well as any targetoid features) were more frequent among iCCAs (*χ*^*2*^ = 15.51 to 91.79, *P* < 0.001 for all), although 21 (19.6%) HCCs presented at least one targetoid appearance. Interobserver agreements were substantial to almost perfect for all imaging features (*κ* = 0.660-1.000, Table 2).

**Table 2:** LI-RADS features and categories of iCCA and HCC in the high-risk population

Features	iCCA (*n* = 107)	HCC (*n* = 107)	*P*	kappa
**Major features**				
Tumor size (cm)^†^	4.7 [3.5, 6.0]	5.0 [3.7, 6.4]	0.234	/
Non-rim APHE	16 (15.0)	88 (82.2)	< 0.001^*^	0.944
Non-peripheral washout	8 (7.5)	84 (78.5)	< 0.001^*^	0.886
Enhancing capsule	14 (13.1)	101 (94.4)	< 0.001^*^	0.785
**LR-TIV**				
Tumor in vein	2 (1.9)	12 (11.2)	0.006^*^	0.788
**LR-M**				
Any targetoid features	91 (85.0)	21 (19.6)	< 0.001^*^	0.897
Rim APHE	79 (73.8)	19 (17.8)	< 0.001^*^	0.830
Peripheral washout	3 (2.8)	1 (0.9)	0.621	0.660
Delayed central enhancement	25 (23.4)	5 (4.7)	< 0.001^*^	0.920
Targetoid diffusion restriction	58 (54.2)	4 (3.7)	< 0.001^*^	0.855
Infiltrative appearance	20 (18.7)	11 (10.3)	0.080	0.706
Marked diffusion restriction	70 (65.4)	76 (71.0)	0.378	0.789
Severe necrosis or ischemia	23 (21.5)	70 (65.4)	< 0.001^*^	0.914
**Ancillary features favoring HCC in particular**				
Non-enhancing capsule	1 (0.9)	3 (2.8)	0.621	0.855
Nodule-in-nodule	1 (0.9)	6 (5.6)	0.119	0.921
Mosaic architecture	1 (0.9)	28 (26.2)	< 0.001^*^	0.761
Fat in mass, more than adjacent liver	0 (0)	29 (27.1)	< 0.001^*^	0.937
Blood products in mass	2 (1.9)	44 (41.1)	< 0.001^*^	0.803
**Ancillary features favoring malignancy**,** not HCC in particular**				
US visibility as discrete nodule	102 (95.3)	105 (98.1)	0.445	/
Corona enhancement	63 (58.9)	49 (45.8)	0.055	0.785
Fat sparing in solid mass	0 (0)	1 (0.9)	0.999	1.000
Iron sparing in solid mass	5 (4.7)	18 (16.8)	0.004^*^	0.951
Restricted diffusion	106 (99.1)	106 (99.1)	0.999	0.798
Mild-moderate T2 hyperintensity	64 (59.8)	57 (53.3)	0.334	0.744
**LI-RADS categories**			< 0.001^*^	/
LR-4	15 (14.0)	1 (0.9)		
LR-5	1 (0.9)	78 (72.9)		
LR-M	89 (83.2)	16 (15.0)		
LR-TIV	2 (1.9)	12 (11.2)		

Unless otherwise indicated, data are numbers of tumors with percentages in parentheses

^†^ Data are median [IQR]

^*^P < 0.05

iCCA, intrahepatic cholangiocarcinoma; HCC, hepatocellular carcinoma; APHE, arterial phase hyperenhancement

Univariate and Multivariate logistic regression results for discriminating iCCA and HCC in the LI-RADS target population were shown in Table 3. The multivariate analysis showed that major features of non-peripheral washout (odds ratio (OR) = 0.135 [95% confidence interval (CI) 0.027, 0.684], *P* = 0.016) and enhancing capsule (OR = 0.007 [95%CI 0.001, 0.076], *P* < 0.001), LR-M feature of severe necrosis or ischemia (OR = 0.156 [95%CI 0.030, 0.826], *P* = 0.029), as well as ancillary feature of blood products in mass (OR = 0.012 [95%CI 0.000, 0.442], *P* = 0.016) were independent imaging findings for discrimination.

**Table 3:** Univariable and multivariable analyses for discriminating iCCA and HCC in the high-risk population

Features		Univariate analysis			Multivariate analysis	
		Odds ratio (95% CI)	*P*		Odds ratio (95% CI)	*P*
**Major features**						
Tumor size (cm)^†^		1.072 (0.957, 1.201)	0.230			
Non-rim APHE		0.038 (0.018, 0.079)	< 0.001			
Non-peripheral washout		0.022 (0.009, 0.052)	< 0.001		0.135 (0.027, 0.684)	0.016
Enhancing capsule		0.009 (0.003, 0.024)	< 0.001		0.007 (0.001, 0.076)	< 0.001
**LR-TIV**						
Tumor in vein		0.151 (0.033, 0.691)	0.015			
**LR-M**						
Any targetoid features		23.292 (11.405, 47.568)	< 0.001			
Rim APHE		13.068 (6.774, 25.208)	< 0.001			
Peripheral washout		3.058 (0.313, 29.873)	0.337			
Delayed central enhancement		6.220 (2.281, 16.961)	< 0.001			
Targetoid diffusion restriction		30.480 (10.468, 88.751)	< 0.001			
Infiltrative appearance		2.006 (0.910, 4.424)	0.084			
Marked diffusion restriction		0.772 (0.433, 1.374)	0.379			
Severe necrosis or ischemia		0.145 (0.079, 0.266)	< 0.001		0.156 (0.030, 0.826)	0.029
**Ancillary features favoring HCC in particular**						
Non-enhancing capsule		0.327 (0.033, 3.195)	0.337			
Nodule-in-nodule		0.159 (0.019, 1.342)	0.091			
Mosaic architecture		0.027 (0.004, 0.200)	< 0.001			
Fat in mass, more than adjacent liver^†^		/	0.999			
Blood products in mass		0.027 (0.006, 0.116)	< 0.001		0.012 (0.000, 0.442)	0.016
**Ancillary features favoring malignancy**,** not HCC in particular**						
US visibility as discrete nodule		0.389 (0.074, 2.048)	0.265			
Corona enhancement		1.759 (1.024, 3.022)	0.041			
Fat sparing in solid mass ^†^		/	0.999			
Iron sparing in solid mass		0.242 (0.086, 0.679)	0.007			
Restricted diffusion		1.000 (0.062, 16.197)	0.999			
Mild-moderate T2 hyperintensity		1.306 (0.759, 2.245)	0.335			

^†^ The odds ratio is + ∞ or -∞ with *P* value of 0.999

iCCA, intrahepatic cholangiocarcinoma; HCC, hepatocellular carcinoma; APHE, arterial phase hyperenhancement

**Fig. 3:**
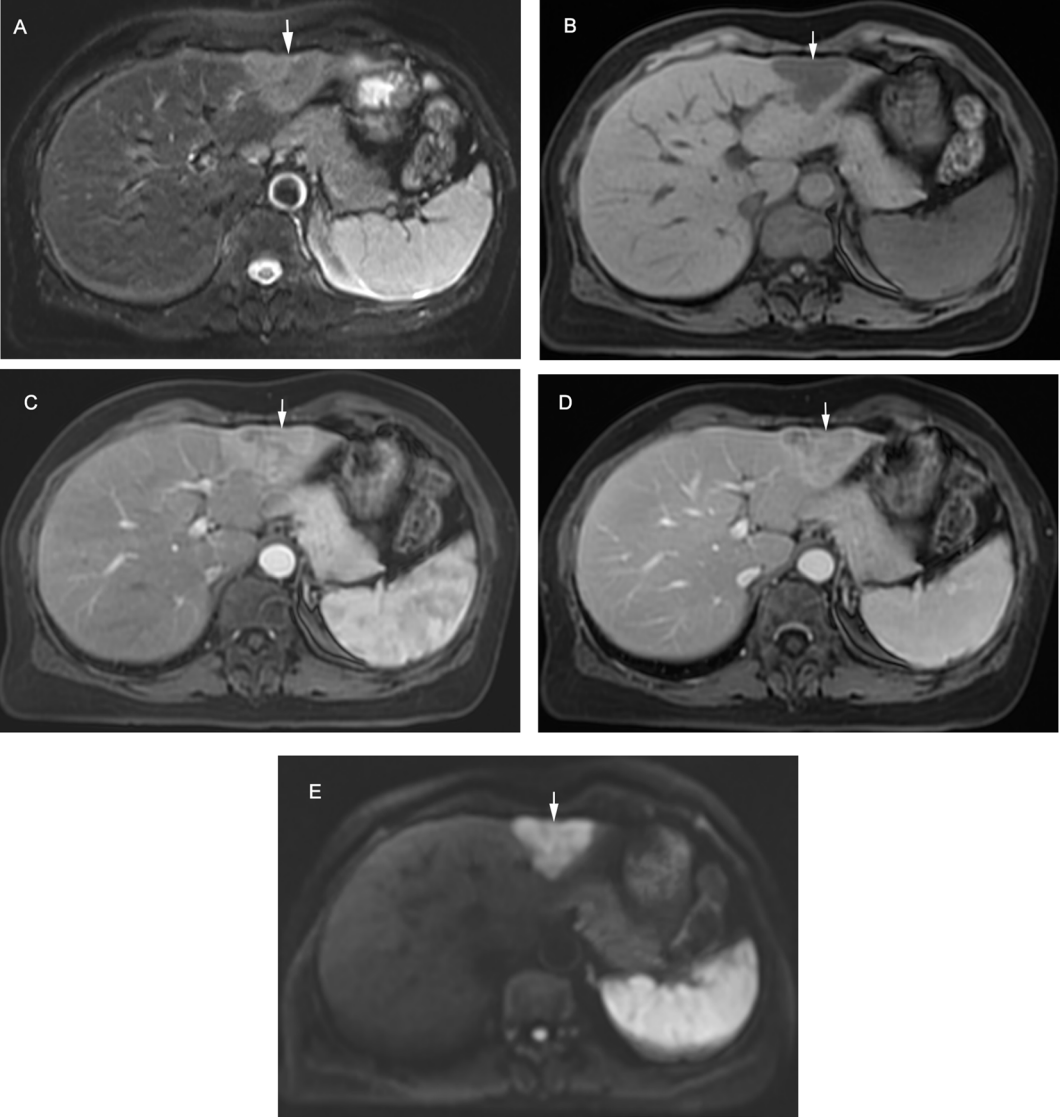
Intrahepatic cholangiocarcinoma in a 59-year-old woman (white arrows). (**A**) T2-weighted image shows a mild hyperintense tumor. (**B**) T1-weighted image shows a hypointense tumor. Contrast-enhanced (**C**) arterial phase image shows non-rim hyperenhancement; (**D**) delayed image shows a washout enhancement pattern with enhancing capsule. (**E)** Diffusion-weighted image (b = 500 s/mm^2^) shows non-targetoid restriction. The lesion is categorized as LR-5

The distribution of the overall LI-RADS categories was significantly different between iCCA and HCC (*χ*^*2*^ = 145.20, *P* < 0.001; Table 2). Most iCCAs were assigned to LR-M category (89/107, 83.2%), the LR-M category had a sensitivity, specificity and accuracy of 83.2%, 85.1% and 84.1% for the diagnosis of iCCA. However, there were 16 (15.0%) lesions miscategorized as probably or definitely HCC (LR-4 or 5), among which only one patient with iCCA was categorized as LR-5 (Fig. 3).

### Diagnostic performance of targetoid imaging appearances for iCCA

The frequencies of targetoid appearances are shown in Table 2. Rim APHE was the most frequent targetoid appearance, and peripheral washout was the least frequent. 91 of 107 (85.0%) iCCAs presented at least one targetoid appearances, and the percentage was 19.6% for HCCs (*χ*^*2*^ = 91.789, *P* < 0.001).

The diagnostic performance of targetoid appearances and the combinations for iCCA in the high-risk patients is presented in Table 4. The best diagnostic performance was achieved when at least one targetoid appearances were required to classify observations as LR-M (AUC = 0.828 [95%CI 0.770, 0.876]), with the highest accuracy of 82.7% and both sensitivity and specificity higher than 80%, superior to any individual targetoid appearance (*z* = 2.614 to 11.986, *P* < 0.001 to 0.009) and the combination of at least 2 (*z* = 3.102, *P* = 0.0019) or at least 3 (*z* = 8.659, *P* < 0.001) targetoid appearances. Adding T2 targetoid appearance did not improve the diagnostic performance for iCCA, although a highest sensitivity of 86.9% could be achieved. The kappa value for T2 targetoid appearance assessment was 0.876 [95%CI 0.798, 0.954].

**Table 4:** Diagnostic performance of targetoid imaging appearance for iCCA in the high-risk population

Targetoid features	AUC (95% CI)	Sensitivity	Specificity	Accuracy
Rim APHE	0.780 (0.719, 0.834)	73.8%	82.2%	78.0%
Peripheral washout	0.509 (0.440, 0.578)	2.8%	99.1%	50.9%
Delayed central enhancement	0.593 (0.524, 0.660)	23.4%	95.3%	59.4%
Targetoid diffusion restriction	0.752 (0.689, 0.809)	54.2%	96.3%	75.2%
At least 1	0.828 (0.770, 0.876)	81.3%	84.3%	82.7%
At least 2	0.738 (0.674, 0.796)	52.3%	95.3%	73.8%
At least 3	0.575 (0.506, 0.642)	17.8%	97.2%	57.5%
All 4^†^	/	/	/	/
T2 targetoid appearance	0.645 (0.577, 0.709)	36.5%	92.5%	64.5%
At least 1 including T2 targetoid	0.818 (0.759, 0.867)	86.9%	76.6%	81.8%

iCCA, intrahepatic cholangiocarcinoma; AUC, area under the curve; 95% CI, 95% confidence interval; APHE, arterial phase hyperenhancement

^†^ No patients presented all 4 targetoid features

### Survival outcome

**Fig. 4:**
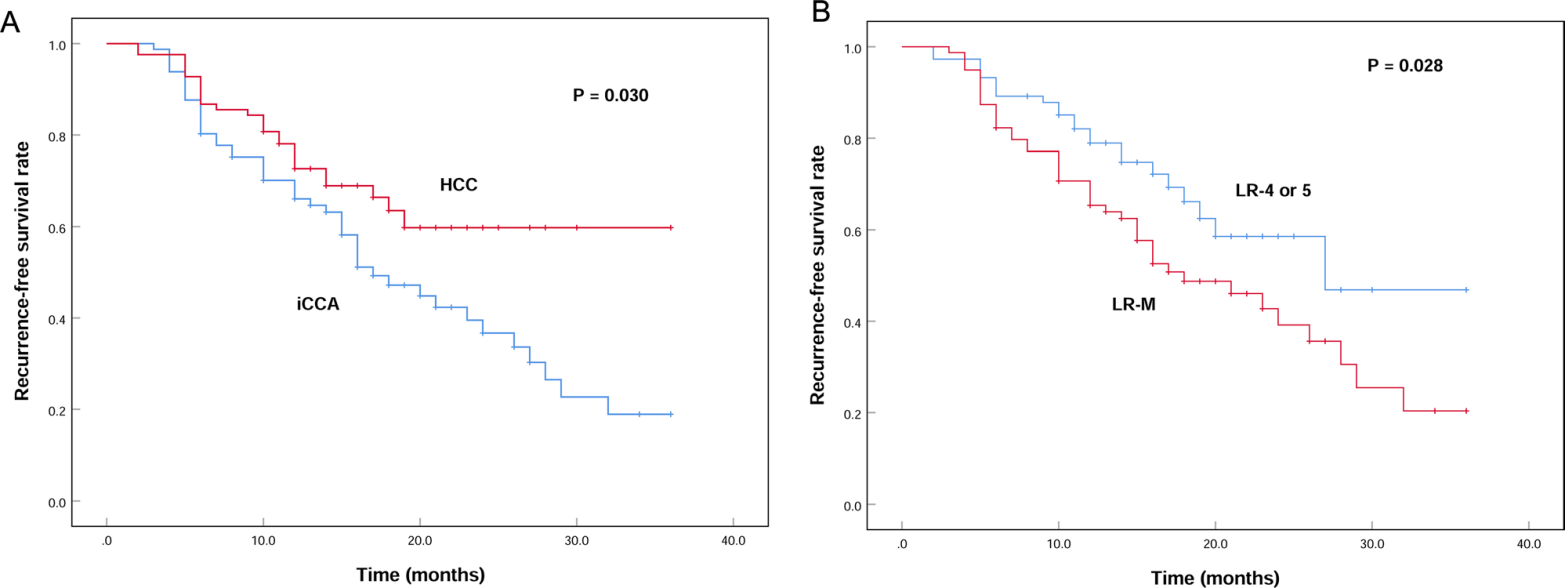
Kaplan-Meier survival curves for recurrence-free survival of (**A**) iCCA and HCC; (**B**) lesions with LR-4 or 5 and LR-M. iCCA, intrahepatic cholangiocarcinoma; HCC, hepatocellular carcinoma

Of the 164 patients with solitary resected iCCA or HCC, who were regularly followed up, 75 had recurrences after surgery. RFS differed according to the pathologic diagnoses, iCCA patients had significantly shorter RFS than HCC patients (log rank *P* = 0.030; Fig. 4A). The 1-year/2-year/3-year RFS rates for iCCA and HCC were 66.1% (95%CI 55.7–76.5%)/36.7% (95%CI 23.6–49.8%)/18.9% (95%CI 5.8–32.0%) and 72.7% (95%CI 62.9–82.5%)/59.8% (95%CI 46.3–73.3%)/59.8% (95%CI 46.3–73.3%), respectively. The LI-RADS categories also showed differences in RFS, patients with LR-M had significantly shorter RFS than patients with LR-4 or 5 (log rank *P* = 0.028; Fig. 4B). The 1-year/2-year/3-year RFS rates for LR-M and LR-4/5 patients were 65.4% (95%CI 54.8–76.0%)/39.2% (95%CI 25.5–52.9%)/20.4% (95%CI 5.1–35.7%) and 78.9% (95%CI 69.3–88.5%)/58.6% (95%CI 43.3–73.9%)/46.9% (95%CI 23.0-70.8%), respectively. At multivariable Cox analysis, only the LI-RADS category (hazard ratio = 1.736 [95%CI 1.046, 2.880], *P* = 0.033) showed an independent correlation with RFS, comparing to variables including pathologic diagnoses as well as age, sex, serum AFP and CA19-9 levels.

## Discussion

Our study revealed that in high-risk patients, iCCA differed from HCC in LI-RADS-based MRI features in general, and the LR-M category facilitated the accurate classification of most iCCAs. In the meantime, LI-RADS-defined risk factors tended to alter the imaging characteristics of iCCAs, leading to a relatively higher prevalence of HCC major features and increased classification as probable HCC. Notably, the LR-5 category demonstrated high specificity in excluding the diagnosis of iCCA. Additionally, among high-risk patients with solitary resected iCCA or HCC, RFS prognosis varied based on both pathologic diagnosis and LI-RADS category, the LI-RADS category showed a stronger correlation with patient outcomes than pathologic diagnosis.

Compared to HCC, iCCA exhibited distinct MRI features in high-risk patients, with features of non-peripheral washout, enhancing capsule, severe necrosis or ischemia, and blood products as independent imaging findings for differentiation, consistent with prior studies [2, 17–19]. The LR-M category enabled the accurate classification of most iCCAs, achieving high specificity and diagnostic accuracy. The targetoid appearance was corresponded to the LR-M category, which is an introduced imaging feature favoring iCCA, reflecting the heterogeneous distribution of cellular and fibrotic components [20]. Most iCCAs were categorized as LR-M when presenting at least one targetoid imaging feature, with the best diagnostic performance and a highest accuracy of 82.7% in our study. Cannella et al. pointed out that the targetoid appearance on T2-weighted images exhibited high specificity for non-HCC malignancies (iCCA and combined hepatocellular-cholangiocarcinoma), which could be added as a valuable diagnostic feature [15]. However, in our high-risk cohort, the T2 targetoid appearance did not significantly enhance the diagnostic performance for iCCA. This discrepancy may be explained by the differences in cohort composition, as Cannella et al. included both iCCAs and combined hepatocellular-cholangiocarcinomas, irrespective of risk factors.

Meanwhile, we noticed that a considerable proportion of iCCAs in high-risk patients exhibited imaging characteristics that overlapped with HCC diagnostic criteria, with about 26% iCCAs presenting at least one major feature and 15% miscategorized as LR-4 or 5. These findings were in line with Cheng et al.’s data, which indicated that at least 20% of iCCAs met the radiologic criteria for HCC (i.e., APHE and/or non-peripheral venous washout) [21]. The proportion may even increase in patients with LI-RADS-defined HCC risk factors [10]. Our finding that the presence of LI-RADS defined risk factors increased the likelihood of iCCAs exhibiting HCC-like imaging features raised concerns of potential misdiagnosis, which would impact therapeutic course and outcomes. However, although 15% iCCAs were miscategorized as probably or definitely HCC, only one iCCA case was categorized as LR-5. Thus, we propose that the LR-5 category is highly specific for ruling out the diagnosis of iCCA in high-risk patients. Given that iCCA is far less prevalent than HCC in high-risk populations, the overall impact on LI-RADS miscategorization for iCCA would likely be modest.

Our results further confirmed that the LI-RADS categorization could serve as a prognostic indicator for postsurgical outcomes in high-risk patients. Although RFS prognosis differed according to both pathologic diagnosis and LI-RADS category, the LI-RADS category was correlated with postsurgical RFS independent of pathologic diagnosis. These findings coincided with prior researches [14, 20], although prior researches primarily focused on patients with cirrhosis. Our study verified this standpoint in a broader high-risk population with solitary resected iCCA and HCC, beyond those with cirrhosis. It is worth noting that most studies, including ours, have emphasized the significance of targetoid LR-M features, particularly rim APHE, which is the most frequent targetoid appearance. The LR-M imaging appearance may be associated with more aggressive tumor behavior and unfavorable prognosis, regardless of pathological type, suggesting a more proactive treatment strategy in clinical practice.

There were several limitations for our study. First, being a retrospective study and the inclusion of only pathologically-proven primary liver cancers by surgery, selection bias could be introduced, our findings require validation in prospective studies with a larger sample size. Second, we aimed to explore the impact of LI-RADS-defined risk factors on the diagnosis and prognosis of iCCA, the proportions of HBV and cirrhotic patients may limit generalization to other populations. Third, as our study focused on iCCA, we enrolled 1:1 matched HCC for comparison following a prior study [14]. Therefore, the ratio in our study may not represent the actual prevalence in the general population. However, this approach created a cohort enriched for iCCA effectively. Lastly, extracellular contrast-enhanced MRI was utilized in this study, precluding an analysis of hepatobiliary-phase LI-RADS features. Nevertheless, increasing evidence has suggested the inferiority of hepatobiliary contrast agent MRI than extracellular contrast agent MRI due to suboptimal arterial phase quality and challenges in the depiction of washout and enhancing capsule on post-arterial phase images [22].

In conclusion, in patients with LI-RADS defined risk factors, although the LR-M category effectively classified most iCCAs, these tumors frequently exhibited HCC major features, leading to miscategorization as probable HCC. However, the LR-5 category remained highly specific for excluding iCCA. Furthermore, for the high-risk patients with solitary resected iCCA or HCC, the LI-RADS category enabled the prediction of postsurgical prognosis, independently from pathological diagnosis.

## Electronic supplementary material

Below is the link to the electronic supplementary material.

Supplementary Material 1

## Data Availability

No datasets were generated or analysed during the current study.

## Abbreviations

iCCA: Intrahepatic cholangiocarcinoma
HCC: Hepatocellular carcinoma
LI-RADS: Liver Imaging Reporting and Data System
T2W: T2-weighted
T1W: T1-weighted
DWI: Diffusion-weighted imaging
RFS: Recurrence-free survival
AUC: Area under the curve
AFP: Alpha fetoprotein
CA19-9: Cancer antigen 19 − 9
APHE: Arterial phase hyperenhancement
OR: Odds ratio
CI: Confidence interval
