# Supplementary material for: Assessment of intrahepatic cholangiocarcinoma with LI-RADS in the high-risk population: MRI diagnosis and postoperative survival
Source: Cancer Imaging. 2025 Mar 26;25:40. doi: 10.1186/s40644-025-00860-6 (PMC11938583; doi:10.1186/s40644-025-00860-6)
Supplement: Supplementary file 1 — Supplementary Material 1 [file 40644_2025_860_MOESM1_ESM.docx]

**Table S1.** MRI sequence parameters

|  | **Parameter** | **T1-weighted IP and OP imaging** | **Contrast enhanced T1-weighted imaging** | **T2-weighted imaging** | **Diffusion weighted imaging** |
| --- | --- | --- | --- | --- | --- |
| Magnetom Aera 1.5T | Repetition time (ms) | 6.87 | 4.36 | 2000 | 2291 |
|  | Echo time (ms) | 2.38/4.76 | 2.0 | 94.0 | 48.9 |
|  | Field of view (mm^2^) | 380×278 | 380×297 | 380×308 | 380×280 |
|  | Matrix | 320×240 | 320×240 | 320×224 | 144×100 |
|  | Section thickness (mm) | 4.0 | 3.0 | 5.5 | 6.0 |
|  | Gap (mm) | 0 | 0 | 1.1 | 1.2 |
| uMR 770 3.0T | Repetition time (ms) | 3.66 | 3.27 | 2000 | 2714 |
|  | Echo time (ms) | 1.2/2.4 | 1.45 | 106.2 | 63.3 |
|  | Field of view (mm^2^) | 400×300 | 400×300 | 380×380 | 380×300 |
|  | Matrix | 168×288 | 288×320 | 256×256 | 128×100 |
|  | Section thickness (mm) | 3.0 | 3.0 | 6.0 | 6.0 |
|  | Gap (mm) | 0 | 0 | 1.2 | 1.2 |

IP, in-phase; OP, opposed-phase
